# Psychosocial Workplace Environments Enabling Sustainable Employment for People with Mental Health Conditions: A Scoping Review

**DOI:** 10.3390/nursrep16030101

**Published:** 2026-03-17

**Authors:** Yoshitomo Fukuura, Yukako Shigematsu, Yumi Mizuochi

**Affiliations:** Department of Nursing, School of Medicine, Kurume University, 777-1 Higashikushiharamachi, Kurume-shi 830-0003, Fukuoka, Japan; shigematsu_yukako@kurume-u.ac.jp (Y.S.); eguchi_yumi@kurume-u.ac.jp (Y.M.)

**Keywords:** mental illness, sustainable employment, psychosocial workplace environment, scoping review, employment retention support

## Abstract

**Background/Objectives:** Research that systematically identifies the components of a psychosocial workplace environment tailored to people with mental illness is limited. This scoping review aimed to map the existing literature and clarify the key concepts of a desirable workplace environment from a psychosocial perspective that enables sustainable employment for people with mental illness. **Methods:** A scoping review was conducted using the Joanna Briggs Institute methodology. Five databases, including PubMed and Scopus, were searched to extract original English-language, peer-reviewed research articles published between 2003 and 2025 on workplace environments for individuals with mental illness. Two independent reviewers screened the records and selected 16 studies using the population, concept, and context framework. Following data extraction, qualitative inductive analysis was conducted for category development. **Results:** Five categories and 17 subcategories were identified as psychosocial workplace environments promoting sustained employment: (1) Growth-supportive environments that leverage individual strengths and promote self-actualization; (2) recognition-affirmative environments that respect individual characteristics and are based on fair evaluation and acceptance of diversity; (3) a low-psychological-strain environment featuring predictability and autonomy; (4) a multilayered support network; and (5) a support environment based on interprofessional collaboration and system utilization. **Conclusions:** Workplace environments supporting the sustained employment of individuals with mental illness appear to involve a multilayered structure integrating self-actualization, predictable and autonomous job design, and comprehensive interprofessional support. The findings provide a preliminary concept map; however, gaps remain in the types and quality of evidence. Future primary research and formal concept analysis are required to validate these components and address existing methodological and contextual gaps.

## 1. Introduction

Employment serves as a crucial opportunity to promote the recovery process of individuals with mental illness. Work not only provides economic stability but also offers elements that are essential for maintaining mental health, such as a sense of purpose, identity, and connection with others [1]. However, the unemployment rate among people with mental disorders is three to seven times higher than that among people without such disorders [2]. Although workplace support is often provided, it focuses on compensating for the perceived weaknesses of individuals with mental illness and may therefore be insufficient [3,4,5,6].

Previous studies have indicated that enabling employees with mental illness to actively engage in the workplace, thereby improving the work environment, is crucial for employment retention [3,6]. Initiatives in which employees with mental illness, colleagues, and employers collaborate to build a supportive workplace environment are likely to contribute to employment retention. However, much of the existing research on the employment of individuals with mental illness has focused on direct support. The traditional “training-then-placement” model is shifting towards employment support models emphasizing “placement-then-training.” Supported Employment (SE) and Individual Placement and Support (IPS) are effective programs for securing employment and improving interpersonal skills [7,8]. Supported employment models have been demonstrated to improve the quality of life of individuals with severe mental illness [9]. Employee assistance programs (EAPs) have been developed to support individual employees by addressing their stress and mental health concerns, thereby contributing to workplace efficiency [10,11]. While such programs often demonstrate certain benefits, numerous challenges remain, particularly in improving employers’ understanding of and engagement with reasonable accommodation and workplace support [7].

The psychosocial workplace environment presents multiple challenges for employees with mental health issues. These include misunderstandings in relationships with supervisors and colleagues, isolation in workplaces with limited knowledge about mental health, and stigma within organizational cultures that treat mental health issues as taboo [12,13,14]. Therefore, improving workplace environments is essential. A psychosocial workplace environment that enables sustained employment may reduce the risk of mental health problems among individuals with mental health challenges [15]. It also contributes to improved quality of life and the discovery of meaningful roles by providing opportunities to recognize and utilize one’s strengths [12,16]. However, the structural components of workplace environments that promote sustainable employment for individuals with mental illness remains unclear. In particular, few reviews have systematically organized the components of psychosocial workplace environments that support sustainable employment for individuals with mental illness, and none have comprehensively presented conceptual definitions or integrated components. Consequently, evidence on workplace environment development remains fragmented, leaving significant research gaps.

Although the workplace is a crucial setting for promoting mental health, research on workplace interventions remains insufficient [17], and the standardization of evaluation methods for workplace improvement is necessary [15]. However, efforts to improve workplace environments have been limited. Improved workplace environments are associated with reduced turnover and greater retention. From the perspective of continuing employment, psychosocial factors are critical for workers’ mental health status, job performance, and recovery [18]. Models also exist in which the workplace provides opportunities for participation in decision making and establishes health management systems to enable a sustained working life [19,20]. Generally, the psychosocial workplace environment comprises elements such as the level of job demands, degree of job autonomy, presence of support from supervisors and colleagues, quality of workplace relationships, and role clarity [8,21]. In this study, a psychosocial workplace environment related to sustainable employment for people with mental illness was operationally defined as one consisting of job accommodations and work arrangements tailored to the characteristics of mental illness, cooperative systems, and interpersonal relationships. However, to date, no academic consensus exists on the definition of a “functional” workplace environment that enables sustainable employment for people with mental illness.

To address this gap, this scoping review aimed to map the existing literature and clarify the key concepts of a desirable workplace environment from a psychosocial perspective that enables sustainable employment for people with mental illness.

## 2. Materials and Methods

This study adopted a scoping review design. The review was conducted following the Joanna Briggs Institute (JBI) methodology [22,23] and reported according to the Preferred Reporting Items for Systematic Reviews and Meta-Analyses—Scoping Review (PRISMA-ScR) statement. The completed PRISMA-ScR checklist is provided in the Supplementary File. The title of this review has been registered in the Open Science Framework (OSF) (https://osf.io/2fymn/overview; accessed on 1 February 2026). Furthermore, this study adhered to the PRISMA checklist as a reporting standard for scoping reviews [24].

### 2.1. Inclusion Criteria

The inclusion criteria were defined using the Population, Concept, Context (PCC) framework based on the components of the review question.

Population: Adults aged 18 years or older but under 60 years who have been diagnosed with any mental disorder.

Concept: Studies describing psychosocially favorable workplace environments that enable individuals with mental disorders to maintain employment.

Setting: Workplace environments where individuals with mental disorders are employed.

### 2.2. Search Strategy

A literature search was conducted on 31 July 2025, using five electronic databases: PubMed, Scopus, Ovid Nursing, Ovid MEDLINE, and ScienceDirect.

The following search terms were used: (“mental disorder” OR “mental illness” OR “mental health disorder” OR “psychiatric disorder” OR “schizophrenia” OR “depression” OR “bipolar disorder” OR “anxiety disorder” OR “post-traumatic stress disorder” OR “obsessive-compulsive disorder” OR “eating disorder” OR “substance use disorder” OR “autism” OR “Attention-deficit hyperactivity disorder (ADHD)”) AND (employment OR “workplace integration” OR “workplace adaptation” OR “reasonable accommodations”) AND (“psychosocial environment of the workplace” OR “psychosocial conditions of the workplace” OR “psychosocial factors” OR “psychosocial factors in the workplace” OR “psychosocial support” OR “psychosocial interventions” OR “psychological workload”).

The complete electronic search strategy for the PubMed database, including all limits used, is presented in Supplementary Table S1.

Two independent reviewers conducted a detailed screening of titles and abstracts and obtained and evaluated full-text articles based on predefined eligibility criteria. Disagreements were resolved through discussion with a third reviewer, who acted as an arbitrator when necessary, ensuring strict adherence to the PCC framework [25].

### 2.3. Study Selection

All records identified through the search strategy were uploaded to Rayyan (Rayyan Systems Inc., Cambridge, MA, USA; https://www.rayyan.ai; accessed 31 July 2025), and duplicates were removed. The platform was used to facilitate blinded screening. Although Rayyan also offers optional AI-assisted features, these were not used in this review. To ensure methodological rigor and transparency, all screening and eligibility assessments were performed manually and independently by the reviewers.

Prior to full screening, a pilot test was conducted using the first 50 records. Two reviewers independently screened these records and achieved an agreement rate of over 90%. Any disagreements during the pilot phase were discussed to clarify the inclusion criteria and refine the screening process. Subsequently, titles and abstracts were screened independently by the two reviewers using a blinded approach.

Studies that did not meet the inclusion criteria were excluded. Full texts were obtained from the relevant papers. A full-text evaluation of the selected literature was performed by three independent reviewers (YF, YS, and YM). Any disagreements arising at any stage of the selection process were resolved through discussions among these reviewers. The reasons for exclusion from the full-text stage were recorded.

The search results and study inclusion process are documented in the PRISMA-ScR flowchart.

### 2.4. Data Extraction and Synthesis

Data were extracted using a structured table aligned with the review objectives and PCC framework, following the JBI Scope Review methodology. The extracted information included title, authors, publication year, country of conduct, purpose, study design, participants, and study outcomes. A concise summary of the included studies and their key findings is provided in the Supplementary File.

The independent reviewers achieved an agreement rate of 95% during the initial data extraction phase. Disagreements were resolved through consultation with a third reviewer.

The findings were derived from a qualitative inductive analysis. Following established methodological recommendations [25,26], an inductive approach was applied. Specifically, data analysis followed the qualitative analysis framework described in a previous study [27]. First, two independent reviewers repeatedly read the extracted data to achieve immersion and coded text segments relevant to the psychosocial workplace environment. Second, using the constant comparative method, the generated codes were grouped into subcategories based on similarities and differences and further abstracted into five final categories. To ensure analytical rigor, inter-rater comparisons and discussions were conducted to resolve disagreements regarding coding or categorization until consensus was reached. Category saturation was determined when no new codes or themes emerged from the data.

## 3. Results

### 3.1. Study Identification and Selection

**Figure 1:**
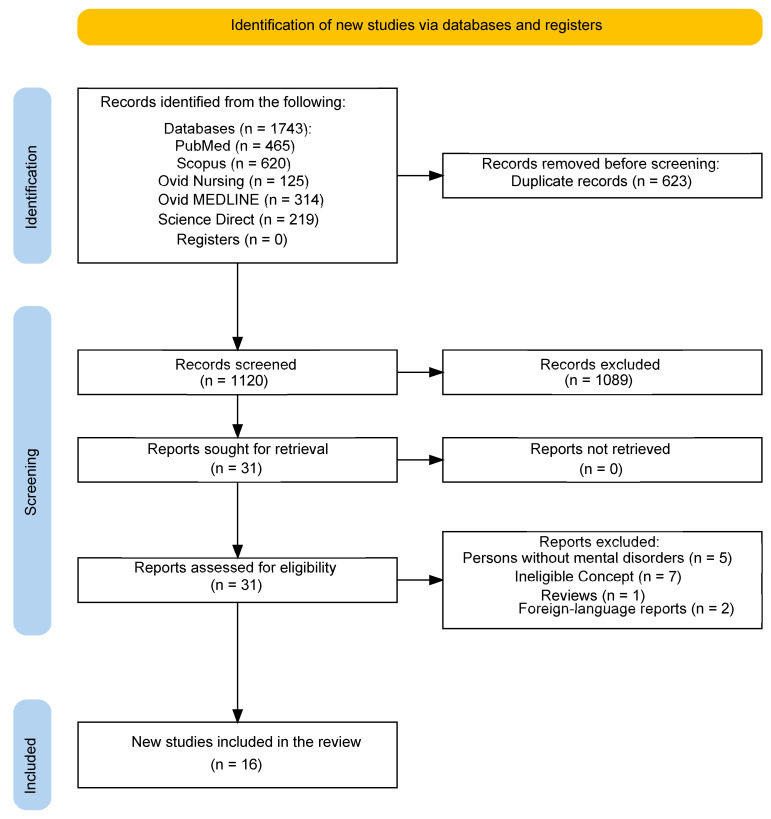
PRISMA-ScR flowchart.

Database searches identified 1743 records. After removing duplicates, 1120 records were screened. Of these, 1089 did not meet the selection criteria, and 31 full-text articles were assessed for eligibility. Following full-text acquisition and a detailed review, 16 studies met the selection criteria, as shown in the PRISMA flow diagram (see Figure 1). Of the 15 excluded full-text articles, seven were classified as “Ineligible Concept.” These studies focused on clinical issues, such as the relationship between inpatient health and work status or associations between symptoms and psychosocial stress, rather than investigating the workplace environment itself.

### 3.2. Characteristics of the Included Studies

The included studies were published between 2003 and 2025, reflecting increased interest in workplace environments related to the employment of people with mental disorders over the past 20 years. These studies were conducted across multiple countries, with most originating from Europe.

Eleven studies employed quantitative designs, including cross-sectional studies [28,29,30,31,32,33], a case–control study [18], a prospective longitudinal pre–post study [34], a descriptive observational study [35], a randomized controlled trial [36], and an observational cohort study [37]. Five studies employed qualitative designs, including qualitative research based on a Constructed Framework for Implementation Research (CFIR) [38], descriptive qualitative methods [39], phenomenological research [8], interpretive phenomenological analysis [40], and a mixed-method approach integrating phenomenological and hermeneutic approaches [41] (refer to Table 1).

### 3.3. Review Findings

This review synthesized evidence from 16 international studies examining workplace psychosocial environments that enable sustained employment of people with mental illness. Five comprehensive categories of workplace environments contributing to employment retention were identified (refer to Table 2).

First, growth-supportive workplace environments that promote self-actualization foster self-efficacy while leveraging individual characteristics and strengths, thereby supporting sustained employment. This category comprises four subcategories: (1) workplaces where employees can utilize their strengths [33], (2) workplaces where employees experience hope and restore self-esteem [8,41], (3) workplaces that provide satisfaction [35,41], and (4) workplaces that allow for self-expression and shared experiences with others [38,40,41]. These findings suggest that environments in which employees not only perform tasks but also re-evaluate their self-worth and experience growth through connections with others are beneficial for sustainable employment.

Second, workplace environments emphasizing recognition that respects individual characteristics highlight the importance of feeling accepted at work, including the acceptance of symptoms and related needs. This category includes two subcategories: (1) workplaces where employees experience a sense of accomplishment and confidence [34,36,39,41] and (2) workplaces that demonstrate an understanding of symptoms and enable diverse work arrangements [29,33,38,39,41]. Specifically, fairness and transparency in promotions and compensation, an understanding of mental health-related characteristics, and culturally sensitive practices were associated with increased self-esteem and trust in the workplace.

Third, a predictable, autonomous, and psychologically low-stress environment refers to one in which employees can work autonomously without excessive burden. This category comprises three subcategories: (1) predictable work environments [33,36,38,41]; (2) work environments that allow discretion and autonomous work [8,18,28,29,31,32,37,41]; and (3) work environments with low psychological burdens [18,30,31,32,36,37,41]. Job discretion enabling active engagement, reduced stress related to workload and interpersonal relationships, and predictable work structures with clear roles and schedules are crucial for sustaining employment while maintaining psychological stability.

Fourth, multilayered support network environments indicate that comprehensive networks comprising diverse supporters are essential for sustaining workers. This category includes five subcategories: (1) supportive workplace environments provided by supervisors [28,32,38,39,40,41]; (2) workplace environments characterized by support and cooperation from colleagues [28,29,30,32,36,37,41]; (3) support environments involving family and community resources [39,40]; (4) workplace environments characterized by safety and security [36,41]; and (5) workplace environments providing practical and life-based support [8]. These subcategories reflect diverse support sources both inside and outside the workplace, suggesting that trust, psychological safety, and practicality (e.g., access to break spaces) form a psychosocial foundation that enables sustained employment.

Finally, workplace environments characterized by interprofessional collaboration and strategic use of institutional systems highlight the role of organizational mechanisms and external expertise in promoting sustainable employment for people with disabilities. This category comprises three subcategories: (1) workplace environments with access to employment support programs [32,35]; (2) workplace environments with ongoing interprofessional support and coordinated involvement [29,35,38,40,41]; and (3) workplace environments utilizing legal protection and social security systems [35,41]. In workplaces implementing programs such as the IPS, participants reported meaningful work experiences and increased self-efficacy. Furthermore, ongoing support from professionals (e.g., mental health social workers, occupational physicians) and the utilization of legal frameworks (e.g., Italian Laws 68/99, 104/92) functioned as an institutional foundation supporting employment stability.

Overall, these findings indicate that the workplace environment necessary for the sustainable employment of people with mental illness is multilayered and comprehensive, requiring the collaborative integration of systems, support providers, and workplace culture.

## 4. Discussion

This scoping review synthesized evidence from 16 international studies on workplace environments enabling sustained employment for individuals with mental illness, and identified key psychosocial factors. These were organized into five categories and 17 subcategories. This section examines the characteristics of psychosocial workplace environments that support sustained employment for individuals with mental illness based on these five categories. Subsequently, the study proposes a refined definition of the psychosocial workplace environment necessary for sustained employment.

### 4.1. Characteristics of Psychosocial Workplace Environments Supporting Sustained Employment for Individuals with Mental Illness

#### 4.1.1. Workplaces Respecting Opportunities for Self-Realization and Individual Characteristics

Across the literature, workplaces where individuals feel respected and are able to utilize their abilities and strengths were repeatedly emphasized as critical foundations for maintaining employment among individuals with mental illness.

Conceptually, viewing employees with mental illness not merely as “recipients of employment support” but as “individuals with growth potential” can form the basis for fostering a positive, psychologically supportive workplace culture. Regarding growth-supportive workplace environments that promote self-actualization, flexible job assignments that utilize individual interests and strengths, along with an understanding of personal values, have been suggested to contribute to increased work motivation and psychological stability.

Furthermore, workplaces that emphasize evaluations based on fairness and respect for diversity have been reported to enhance workplace trust by treating employees equally without discrimination or bias. Transparency in evaluation systems, equitable opportunities for promotion and compensation, and the acceptance of cultural and psychological diversity are highly likely to foster a sense of recognition and self-esteem among employees with mental illness. Consistent with prior research, self-efficacy (confidence in one’s ability to perform tasks) is a key mediating factor that enhances both sustained employment and psychological well-being [42]. Furthermore, assigning “meaningful work” that reflects employees’ values, interests, and abilities rather than merely assigning simple tasks as acts of consideration has been identified as essential for generating positive psychological effects [43,44].

In summary, growth-supportive workplace environments that promote self-actualization and recognition-based workplace environments that respect individual characteristics can be interpreted as the psychological foundation for employment retention, as they enable a sense of meaning in work and respect for humanity in the workplace.

#### 4.1.2. Workplaces Providing Predictability and Discretion

For individuals with mental health conditions to work stably in the long term, a workplace environment that provides predictability regarding job content and workload and allows them to actively engage with discretion is essential. An analysis of psychologically low-stress environments characterized by predictability and autonomy indicated that excessive workload, sudden changes, and ambiguous instructions increase psychological stress and can become risk factors for turnover.

Conversely, workplaces with clearly structured tasks and flexible working arrangements tailored to employee health conditions and preferences have been shown to reduce stress and enhance self-efficacy. Previous research indicates that, in highly autonomous environments, employees can adjust their work to their health status without requiring individual approval from supervisors. This flexibility allows them to maintain productivity without formally requesting workplace accommodation, which may reduce the need for repeated formal accommodation requests, thereby contributing significantly to continued paid employment [45]. Furthermore, the ability to autonomously adapt to work demands may reduce the need to request accommodation and alleviate the psychological conflict associated with disclosing mental illness.

A sense of control over work (autonomy) enhances self-management capabilities and psychological stability. Systems that enable employees to adjust work processes through proactive communication with supervisors and colleagues create environments in which employees can continue to work with greater peace of mind. Strengthening job control is considered essential for promoting mental health, and expanding discretion within the workplace environment is regarded as critical for occupational mental health [46].

Furthermore, as the findings indicate, role clarity is critical for supporting predictability. When employees accurately understand what is expected of them and rely on a predictable work structure, trust in the workplace culture is strengthened, potentially providing a psychological foundation that supports continued employment. Notably, the optimal balance between predictability and autonomy may vary depending on the diagnostic category and symptom severity. While the included studies supporting this category involved participants with diverse conditions (e.g., schizophrenia, mood disorders, anxiety disorders), the impact of autonomy might differ. For individuals with severe symptoms or cognitive impairments—often associated with acute phases of schizophrenia or severe depression—excessive discretion may lead to ambiguity and increased anxiety. In such cases, a highly structured environment with clear predictability may be prioritized over high autonomy. Therefore, discretion provision should not be uniform but rather tailored to individuals’ functional levels and recovery stages.

#### 4.1.3. Workplace Environments with Multilayered, Institutional Support Systems for Employment Retention

To ensure that the employment of individuals with mental illness is long-term and stable rather than temporary, building a multilayered support network environment is necessary, involving multiple supporters both inside and outside the workplace, including the family. The reviewed studies emphasized the importance of mechanisms in which supervisors and colleagues provide daily support, while family, healthcare providers, community support organizations, and specialists collaborate to accompany workers and monitor their employment situation over the long term.

As a complementary element to this network, institutionalizing the workplace itself as a mutual-support community may be effective. Specifically, implementing peer support to prevent social isolation and establishing institutional mental health support, such as counseling, reportedly contribute to fostering employee security and maintaining productivity [47].

Furthermore, evidence regarding support environments based on interprofessional collaboration and system utilization indicates that combining the active use of disability employment systems, vocational transition services, and occupational health systems with coordinated collaboration among specialists, such as mental health social workers, case workers, and occupational physicians, can build a more comprehensive support structure. In other words, strengthening the organization’s overall capacity is crucial to support sustainable employment rather than place the burden solely on the individual capabilities of employees with mental illness. Ensuring the effectiveness and fairness of the return-to-work process requires indispensable interprofessional collaboration and the establishment of a cooperative framework through continuous and constructive dialogue among specialists [48].

Collectively, these multilayered supports function as safety nets that respond flexibly to fluctuations in health status and life challenges, enabling sustained employment through integrated support in both work and daily life.

### 4.2. Proposing a Tentative Concept Map for Workplace Environments Supporting the Sustained Employment of Individuals with Mental Illness

Based on the integrated findings, we propose a tentative concept map as a starting point for future investigation: sustainable employment for individuals with mental illness may be supported by workplace environments that integrate recognition of individual characteristics and self-actualization, predictable and autonomous work arrangements, and coordinated, multilayered support systems.

It is important to emphasize that this map is preliminary, derived solely from the 16 studies included in this review, and should not be considered validated. Its applicability across diverse diagnostic groups, cultural contexts, and employment types remains untested, and the types and quality of evidence across studies varied considerably. Formal concept analysis and prospective studies are needed to examine whether and how these components interact to promote sustained employment.

### 4.3. Implications for Future Research

This scoping review reveals several gaps in the existing evidence base. First, regarding study design, most included studies (11 of 16) employed cross-sectional or qualitative designs, with only one randomized controlled trial and two longitudinal studies. As noted in methodological guidance on scoping reviews, such designs are appropriate for mapping the breadth of evidence but are insufficient for establishing causal relationships between psychosocial workplace factors and sustained employment outcomes [25,26]. Future research may consider incorporating longitudinal and intervention-based designs to examine how psychosocial environmental components change over time and whether modifications to these components predict employment retention.

Second, a geographic gap was observed: most included studies were conducted in European countries (Sweden, Germany, the Netherlands, Denmark, the United Kingdom, Norway, Italy, and France), with only three from Asia (two from Japan and one from South Korea) and one from Turkey. There is a notable absence of evidence from low- and middle-income countries, as well as from East and Southeast Asian, African, and Latin American contexts. Future research may need to investigate whether the concept map proposed here holds across different cultural, institutional, and labor market settings.

Third, a diagnostic gap was identified. Although this review included studies across a range of mental health conditions (e.g., schizophrenia, mood disorders, anxiety disorders, ADHD, autism spectrum disorder), no study directly compared psychosocial workplace environment components across diagnostic categories. Given that symptom profiles and functional impairments differ substantially across diagnoses, it remains unclear whether the five categories identified in this review are equally applicable across populations. Future research would be warranted to examine the differential impact of psychosocial workplace factors by diagnosis, symptom severity, and stage of recovery.

Fourth, a conceptual gap was identified. Although this review maps five categories of psychosocial workplace components, no academic consensus exists on how these components are defined or measured. Standardized instruments and validated conceptual definitions are lacking. Formal concept analysis using established frameworks [49] and Delphi consensus studies [50,51] involving people with lived experience, employers, and occupational health professionals could help validate and operationalize the tentative map proposed here.

Fifth, an environmental context gap was identified. While the review period encompasses a time of significant transformation in working styles, the impact of contemporary arrangements—such as remote or hybrid work—on the sustainable employment of people with mental illness remains underexplored. Future research is encouraged to evaluate how emerging flexible work models and digital communication tools affect psychosocial well-being and the need for reasonable accommodations.

### 4.4. Limitations

While this scoping review systematically organized psychosocial environmental factors in workplaces that support sustainable employment for individuals with mental illness, several limitations can be identified. First, the search was limited to five major electronic databases and did not include gray literature (such as dissertations and government reports) or unpublished research. This exclusion of gray literature may have omitted practical guidelines or policy-level reports that are not published in academic journals, potentially limiting the review’s comprehensiveness in terms of non-academic interventions. Furthermore, restricting the scope to English peer-reviewed original articles may have underestimated the findings reported in other languages, including Japanese, potentially introducing geographical and cultural biases into the evidence base.

Second, while this review broadly defined individuals with mental illness to include diverse diagnoses, specific workplace needs may vary, depending on symptom severity, functional level, and occupational demands. Therefore, an integrated classification approach may not be able to fully distinguish the specific environmental requirements associated with each diagnostic category. Further consideration is required regarding the differences in environmental factors based on occupation and employment type.

Third, because of the nature of the scoping review, no formal assessment of the study quality (risk of bias) was conducted, making it difficult to judge the strength of the evidence for the extracted elements. Furthermore, many of the included studies were cross-sectional or qualitative and failed to establish causal relationships between specific factors and sustained employment. Additionally, inconsistent definitions and measurement methods for “the psychosocial workplace environment” across studies posed constraints when attempting to integrate or redefine the concept.

Fourth, the review period (2003–2025) encompassed a time of significant transformation in workplace environments, including the proliferation of information and communication technologies and the expansion of remote work.

However, the impact of these new ways of working on the sustainable employment of people with mental illness may not have been fully captured in the reviewed literature. Consequently, the findings may predominantly reflect traditional workplace models, and the long-term impacts of post-pandemic work arrangements (e.g., hybrid work) may not be fully represented.

## 5. Conclusions

Based on a synthesis of 16 international studies, this scoping review mapped five categories and 17 subcategories characterizing psychosocial workplace environments that enable sustainable employment for individuals with mental illness. The results suggested that the sustained employment of individuals with mental illness may be supported by psychological safety based on self-actualization and fair treatment, flexible job structures that provide predictability and discretion, and comprehensive support systems underpinned by organizational support and multilayered networks. These elements appear to be interrelated and may form a psychosocial foundation for sustained employment by potentially enhancing self-efficacy and trust in the workplace. Based on the findings, the key concepts identified in this review—growth-supportive environments, recognition-based support, predictability and autonomy, and multilayered interprofessional systems—provide a preliminary concept map that warrants further empirical investigation. Important gaps remain: most evidence derives from cross-sectional and qualitative studies conducted in European contexts, and no included study directly compares psychosocial environment components across diagnostic categories or employment types. Future primary research and formal concept analysis are needed to clarify these components and evaluate their role in sustainable employment.

## Figures and Tables

**Table 1:** Characteristics of included studies and identified psychosocial workplace factors.

No	Title	Author(s)	Year	Country	Objective/Aim	Study Design	Participants/Population	Conclusion(s)/Key Findings
1	Mental wellbeing and psychosocial working conditions of autistic veterinary surgeons in the UK [28]	Smits, F.; Houdmont, J.; Hill, B.; Pickles, K.	2023	United Kingdom	To explore the association between mental health and psychosocial working conditions among veterinarians with autism spectrum disorder (ASD) in the UK	Exploratory cross-sectional study	Of 106 responses, 85 were analyzed. Inclusion criteria were either a formal ASD diagnosis or self-identification as having ASD. Twenty-one (24.7%) reported a formal diagnosis, while 64 (75.3%) self-identified as autistic. The majority of participants were female (84.7%), with companion animal practice being the primary specialism (75.3%).	Veterinarians with autism were found to face markedly poorer mental health and lower-quality workplace environments compared with veterinarians without autism and general workers. Specifically, psychosocial working conditions—namely job autonomy and role clarity—accounted for 44% of the variance in mental health status. These findings suggest that workplace interventions supporting the mental health of this group should focus on enhancing job autonomy and clarifying roles.
2	Working conditions, psychosocial environmental factors, and depressive symptoms among wage workers in South Korea [32]	Sohn, M.; Choi, M.; Jung, M.	2016	South Korea	To investigate the impact of working conditions and psychosocial factors on depressive symptoms	Cross-sectional study	Sample comprised 4095 individuals (53.5% male, 46.5% female). Standard workers accounted for 85.3%, while atypical (precarious) workers constituted 14.7%.	Workers in unstable employment arrangements (e.g., non-regular employment) or with irregular working hours had a 1.66-fold higher risk of depressive symptoms compared with workers in standard employment arrangements. Furthermore, individuals exposed to high job demands (e.g., heavy workload or high emotional labor) who received low social support demonstrated a 1.61-fold increased likelihood of exhibiting depressive symptoms compared with those receiving high social support. This suggests that low social support amplifies the association between high job demands and depression. Overall, this study recommends intervention programs that strengthen workplace mental health education and social support to reduce workplace depression.
3	Psychosocial working conditions and the risk of depression and anxiety disorders in the Danish workforce [18]	Wieclaw, J.; Agerbo, E.; Mortensen, P.B.; Burr, H.; Tuchsen, F.; Bonde, J.P.	2008	Denmark	To examine the association between psychosocial working conditions and the risk of depressive and anxiety disorders	Population-based nested case-control study	This study included 14,166 cases aged 18–65 years with a first diagnosis of depressive disorder (F30–39) or anxiety disorder (F40–48), matched by sex and age, and a control group without psychiatric hospitalization history. Approximately 62% were female; 67% of cases were diagnosed with anxiety disorders, while 33% were diagnosed with depressive disorders.	Risk patterns for mental disorders differed by gender. For example, among men, low job autonomy was associated with increased risk of anxiety disorders, whereas among women, high emotional strain was associated with increased risk of depression. Overall, these findings highlighted gender differences in risk patterns and suggested that evidence regarding the causal relationship between mental disorders and workplace exposures within the demand-control-support model (comprising job demands, job control including autonomy, decision-making authority, skill discretion, and the combination of high demands and low control) remains inconsistent. This underscores the need to reconsider research designs.
4	An integrated mental health and vocational intervention: A longitudinal study on mental health changes among young adults [34]	Liljeholm, U.; Argentzell, E.; Bejerholm, U.	2020	Sweden	To evaluate 12-month mental health changes among young adults participating in the Södertälje Supported Employment and Education (SSEE) integrated mental health and employment support model	Prospective longitudinal pre-post (12-month) design	Included 42 young adults aged 18–29 years who were using mental health services, were motivated to work, and had been diagnosed with a mood disorder (major depressive disorder or bipolar disorder).	Statistically significant improvements in quality of life and activity participation were observed among young adults with mood disorders. Depressive symptoms and empowerment scores also showed favorable trends, suggesting this integrated service can support both individual recovery and clinical remission. In conclusion, interventions incorporating early vocational support, such as the SSEE model, are recommended, as they are essential for improving mental health and promoting social participation among young adults.
5	Psychosocial factors and colleagues’ perceptions of return-to-work opportunities for workers with a psychiatric disorder: a Japanese population-based study [29]	Eguchi, H.; Wada, K.; Higuchi, Y.; Smith, D.R.	2017	Japan	To examine the association between psychosocial factors in the workplace (e.g., job autonomy, workplace social support) and colleagues’ negative perceptions that opportunities for return to work are not provided to colleagues with work limitations owing to mental disorders	Cross-sectional study	3710 Japanese workers aged 20–69 (1855 men, 1855 women) were recruited, evenly allocated across 10-year age groups. Healthcare professionals were excluded.	A survey of 3710 Japanese workers found that over half held negative perceptions regarding return-to-work (RTW) opportunities for those with psychiatric disorders. Psychosocial factors, specifically low social support and low job control, were strongly associated with these negative attitudes. Lack of prior experience and smaller company size also contributed. The study concludes that improving workplace environments by enhancing support and autonomy is essential for successful reintegration.
6	Employment and Social Security/Insurance among patients affected by mental disorders in Italy: A descriptive multi-center study [35]	Ventriglio, A.; Latorre, M.; Calabretta, M.A.; Cuomo, A.; Di Gioia, I.; Ducci, G.; Ghio, L.; Mallozzi, A.; Politi, P.; Suma, D.; Tarricone, I.; Valentini Gravinese, G.; Vita, A.; Working Group E.; Bellomo, A.	2023	Italy	To descriptively characterize employment rates, social security systems, and rehabilitation interventions for people with severe mental illness in Italy	Descriptive observational study	This study included 737 participants aged 18 to 70 years. The main diagnostic groups were psychotic disorders (schizophrenia spectrum: 34.1%), mood disorders (bipolar disorder/depression: 40.9%), personality disorders (11.2%), anxiety disorders (2.84%), and other diagnoses (10.9%). The average follow-up period at regional mental health centers was 9.86 years.	A study involving 737 patients revealed an employment rate of merely 35.8% within the sample. Individuals with psychotic disorders (schizophrenia spectrum disorders), in particular, exhibited higher unemployment rates, more severe occupational impairment, and greater utilization of employment support programs and rehabilitation services. These findings underscore the importance of psychosocial support and interventions for this population within a recovery-oriented treatment framework.
7	Organizational changes and depression: The mediating role of psychosocial work exposures in the SUMER study [30]	Niedhammer, I.; Quatrevaux, M.; Bertrais, S.	2025	France	To examine the association between various organizational changes and depression (PHQ-9) in a nationally representative sample of employees in France To assess the mediating role of psychosocial occupational exposures in this association	Cross-sectional study	The study included 25,977 employees (14,682 men and 11,295 women) who completed self-administered questionnaires.	Experiences of organizational change, particularly frequent change, were associated with increased risk of depression among employees. This association was primarily mediated by psychosocial factors, including high psychological strain, low self-esteem, low job security, workplace bullying, and ethical conflict, suggesting that these are key targets for prevention. Overall, strengthening prevention measures focused on work organizations and psychosocial workplace environments is essential to reduce depression in the working population.
8	How do employees currently admitted to acute psychiatric inpatient units rate their psychosocial working conditions with the Copenhagen Psychosocial Questionnaire (COPSOQ) [36]	Brucks, A.; Lang, A.; Blank, D.; Lincke, H.J.; Siafis, S.; Brieger, P.; Hamann, J.	2023	Germany	To explore how COPSOQ (Psychosocial Work Environment) assessments correlate with clinical characteristics and symptom severity in working adults hospitalized for acute mental illness, and identify potential clinical factors explaining observed differences by comparing these assessments with large-scale benchmark data (FFAW) from the general working population in Germany	A multicenter cluster randomized controlled trial evaluating the effectiveness of case management interventions by return-to-work (RTW) specialists in adults hospitalized for mental illness who continued working	A total of 268 hospitalized patients enrolled in the RETURN study (COPSOQ data providers 229–265). The mean age was approximately 41 years, with 59% female participants. Diagnoses included mood disorders such as depression (67%) and psychotic disorders such as schizophrenia (24%). Employment status was full-time for 71% and part-time for 29%.	Although psychiatric inpatients did not necessarily rate their workplace environment as worse than that of the general workforce, they scored significantly lower on the COPSOQ “Impact” domain (covering burnout symptoms and general health). This suggests that a complex relationship exists between mental health and work-related perceptions, indicating that clinical factors (particularly depressive symptoms) significantly influence the assessment of work-related impact.
9	Experiences of participating in a problem-solving intervention with workplace involvement in Swedish primary health care: a qualitative study from rehabilitation coordinator’s, employee’s, and manager’s perspectives [38]	Karlsson, I.; Kwak, L.; Axén, I.; Bergström, G.; Bültmann, U.; Holmgren, K.; Björk Brämberg, E.	2023	Sweden	To explore the experiences of employees participating in workplace-based problem-solving interventions (PSI-WPI) within primary care during sick leave due to common mental disorders (CMDs) and to identify factors promoting or hindering participation	Qualitative research (CFIR: Consolidated Framework for Implementation Research)	Participants: Interviews conducted with 29 individuals: Rehabilitation Coordinators (RC) (n = 8), Employees (n = 13), Line Managers (n = 8).	Data collected from rehabilitation coordinators, employees, and managers indicated that the intervention provided a structured framework supporting dialogue building and the identification of problems and solutions. However, it also became clear that successful participation required good relationships between stakeholders, and that the time- and labor-intensive nature of the intervention could act as a barrier.
10	Effect Modification by Attention Deficit Hyperactivity Disorder (ADHD) Symptoms on the Association of Psychosocial Work Environments With Psychological Distress and Work Engagement [31]	Nagata, M.; Nagata, T.; Inoue, A.; Mori, K.; Matsuda, S.	2019	Japan	To examine whether ADHD symptoms mediate the relationship between psychosocial workplace environments (job demands, job autonomy, workplace social support) and health outcomes (psychological distress and work engagement)	Cross-sectional study using self-administered questionnaires were distributed to employees of a Japanese pharmaceutical company. Multiple regression analyses, stepwise adjusted for age, gender, job type, and educational attainment, examined interaction terms (“ADHD symptoms × workplace environment”) and conducted simple slope analyses.	Questionnaires were distributed to 4738 employees, with responses obtained from 2791 (response rate: 59.8%). After excluding cases with missing data, 2693 participants were included in the analysis. The mean age was 42.8 years, with women comprising 27.8% of the sample. The prevalence of ADHD symptoms (ASRS screener positive) was 5.9% (n = 159).	This cross-sectional study of employees at a Japanese pharmaceutical company revealed that job autonomy and social support significantly influence the psychological distress of individuals with ADHD symptoms. However, no evidence was found to suggest that ADHD symptoms modify the relationship between workplace environment and work engagement. This study aimed to provide insights contributing to the creation of better workplace environments for employees with ADHD symptoms.
11	Depressive and anxiety disorders on-the-job: the importance of job characteristics for good work functioning in persons with depressive and anxiety disorders [37]	Plaisier, I.; de Graaf, R.; de Bruijn, J.; Smit, J.; van Dyck, R.; Beekman, A.; Penninx, B.	2012	The Netherlands	To examine the impact of job characteristics (occupation, psychosocial working conditions, and working hours) on absenteeism and at-work performance among workers with depressive and/or anxiety disorders and healthy workers To investigate interactions between job characteristics and depressive/anxiety disorders, exploring whether specific job characteristics are particularly beneficial for workers with these disorders	Multisite naturalistic observational cohort study	Sample: 2981 participants aged 18–65 years, including individuals with current and remitted depressive/anxiety disorders, high-risk groups, and healthy controls. Of 1726 participants in paid employment (>8 h/week), 1522 completed the psychosocial work environment questionnaire (536 men, 986 women). No current disorder: 393 (25.8%) Current (past 6 months) depressive and/or anxiety disorder: 767 (50.4%) Current depression only: 202 (26.3%) Current anxiety disorder only: 276 (36.0%) Current comorbid depression + anxiety: 289 (37.7%) Remitted (past diagnosis, none in past 6 months): 362 (23.8%)	High job autonomy, sufficient workplace support, and shorter working hours were associated with reduced absenteeism and improved work functioning not only among individuals with mental health problems but across the entire workforce. This suggests that a favorable workplace environment is a key determinant of better work functioning, irrespective of the mental health status. Overall, this study indicates practical intervention opportunities to mitigate workplace difficulties for employees with mental disorders.
12	Shortlists of workplace support for employees with autism: A freelisting study in the UK [33]	Petty, S.; Eccles, N.; Tunstall, L.; Richardson, H.	2023	United Kingdom	To understand the actual workplace accommodations implemented for employees with autism spectrum disorder (ASD) in UK workplaces	Cross-sectional study	Total participants: 98 (survey distributed to all employees within the organization). Percentage reporting having a colleague diagnosed with ASD: 23% Percentage who answered “don’t know” regarding whether they had a colleague with ASD: 14% Percentage reporting personal experience with ASD (friends, family, etc.): 85% Percentage who had received workplace training on ASD: 27% Percentage of those who identified themselves as autistic: 15% Percentage who answered that they were unsure whether they were autistic: 6%	Respondents recognized that employees with autism possess many strengths, such as attention to detail, innovative thinking, and providing different perspectives, while noting that noisy environments are a common challenge. Crucially, however, the majority reported that few specific accommodations to support employees with autism are implemented in the workplace, or that they are unaware of such accommodations. This survey highlights the gap between legal requirements and workplace practice, emphasizing the need to invest in more inclusive and supportive work environments.
13	Views of individuals diagnosed with schizophrenia on working life: A qualitative study [39]	Duman, Z.Ç.; Tuncer, G.Z.; Sarı, A.; Alptekin, K.	2021	Turkey	To elucidate the lived experiences of individuals diagnosed with schizophrenia who possess practical work experience	Descriptive qualitative research	Interviews were conducted with 11 participants diagnosed with schizophrenia. The average age was 47 years, with an average duration of illness of 21 years. Participants comprised 9 male and 2 female individuals, including 2 married and 9 single individuals. Employment status was as follows: 5 full-time, 2 part-time, and 4 previously employed but currently unemployed. Educational attainment was as follows: primary school graduate (n = 2), junior high school graduate (n = 2), senior high school graduate (n = 4), university graduate (n = 3). Ten lived with relatives, and one lived alone.	Data were collected through semi-structured interviews with 11 participants, yielding four main themes: “Employment as part of daily life,” “Facilitating factors,” “Barriers,” and “Suggestions and expectations.” In conclusion, employment is recognized as an important element of life for individuals with schizophrenia. Addressing barriers such as stigma and medication side effects, alongside the integration of support programs by mental health professionals, was found to be essential for social participation and recovery.
14	“Without IPS I Think I Would Really Fall Apart”: Individual Placement and Support as Experienced by People With Mental Illness-Phenomenological Peer Research Study [8]	Borowska, M.A.; Ørjasæter, K.B.; Borg, M.; Stenvall, B.; Silbermann, A.; Rinaldi, M.; Killackey, E.; Mykletun, A.; Moe, C.	2025	Norway	To gain a deeper understanding of how the IPS phenomenon is experienced in participants’ daily lives, with a particular focus on the relationship between Employment Specialists (ES) and IPS participants within organizational and social contexts. This study also aimed to address identified gaps in qualitative research examining the relationship between ES and participants, including organizational and social contexts	Qualitative phenomenological study	Participants: Ten IPS service users (six women, four men, aged 25–54) utilizing mental health services in Northern Norway where IPS services are provided. Employment status at initial interview: seven job-seeking, one full-time employed, one part-time employed, one temporary employed.	For participants, IPS functioned as an “anchor” beyond mere job support, providing a “safety net” throughout the employment process. This fostered transformative relationships, strengthened confidence and self-worth, and enabled participants to feel “seen as a person, not just a patient.” A key finding was the frequent observation of employment specialists operating beyond the formal scope of vocational support to address participants’ diverse needs, which fragmented welfare services could not adequately meet. This raises questions about adherence to the model while highlighting the necessity for enhanced coordination between welfare and healthcare services.
15	Psychosis and the experience of employment [40]	Nithsdale, V.; Davies, J.; Croucher, P.	2008	United Kingdom	To explore the employment experiences of individuals who have experienced psychosis, including schizophrenia, and to gain a deeper understanding of the experiences of those who have successfully secured competitive employment	Interpretative phenomenological analysis (IPA)	Eight participants (four male, four female). Four had schizophrenia, four had other psychotic disorders.	Substantive research focused on the employment experiences of individuals who had experienced psychosis, including schizophrenia. By examining participants’ lived experiences of securing and maintaining paid employment, the researcher identified the following key themes: interpersonal support, experiences of discrimination, and the conflict between job demands and symptom management. Stigma and prejudice in the workplace, and concerns about relapse, emerged as particularly significant challenges for these individuals.
16	The meaning of the working cooperative for persons with long-term mental illness: a phenomenological study [41]	Gahnström-Strandqvist, K.; Liukko, A.; Tham, K.	2003	Sweden	To investigate the phenomenon of participation in cooperatives and to gain a deep understanding of cooperatives as a “living lifeworld”	An empirical phenomenological psychology (EPP) methodology combining phenomenological and hermeneutic approaches	Participants: 18 individuals: 14 with schizophrenia, 4 with other diagnoses (borderline personality disorder, anxiety/phobia, depression). Ten participants were female.	Participation in the cooperative was described as fostering a sense of “ordinary life” characterized by meaning, joy, belonging, and a sense of accomplishment throughout daily existence. Furthermore, it was suggested that professional engagement, relationship building, and social support within the cooperative are essential elements supporting participants’ well-being.

**Table 2:** Results of qualitative descriptive analysis.

Categories (5)	Subcategories (17)	Content	References
Growth-supportive workplace environment that promotes self-actualization	A workplace environment that enables individuals to leverage their strengths	Attention to detail: Being meticulous, paying close attention to detail and checking details; strengths in data-inputting	[33]
		Contributing innovative and creative ways of thinking	[33]
		Enhanced focus and ability to concentrate without getting distracted; getting absorbed in tasks	[33]
	A workplace environment that enables perceived restoration of hope and self-esteem	“She Employment Specialist (ES) is very curious about me, and she sees those healthy parts of me. She is not just seeking those healthy parts; she is able to find them, accept them, and relate to them.” ESs transcend diagnostic labels, acknowledging participants as persons, guiding them to unearth and amplify their innate abilities.	[8]
		First, an ES advocates for the possibilities, positive outcomes, and new beginnings within a safe environment rather than emphasizing barriers. One participant reflects on his path and the role of the ES guiding him through adversities, sharing a lived experience that resonates with moving beyond past failures.	[8]
		Having social goals	[41]
		Building skills and confidence	[41]
	A workplace environment that provides a sense of satisfaction	Finding meaning in employment through Individual Placement and Support (IPS)	[35]
		A rich yet ordinary workplace environment	[41]
		Not feeling intense loneliness	[41]
		Gaining a sense of accomplishment	[41]
		Having one’s achievements recognized by others	[41]
		A workplace environment enabling more active involvement in family life	[41]
	A workplace environment that allows self-expression and sharing experiences with others	Being able to articulate personal issues and proactively choose solutions (During interviews, supportive questions helped organize thoughts and provided motivation to move forward)	[38]
		Disclosing one’s condition has a positive impact	[40]
		Being able to share experiences with others	[41]
Recognition-affirming workplace environment that respects individual characteristics	A workplace environment that enables a sense of accomplishment and confidence	Appropriate evaluation and recognition (self-esteem), fair promotion opportunities, employment stability	[34]
		High trust and fairness	[36]
		Not receiving unfair treatment	[36]
		High evaluation	[36]
		Receiving rewards for effort	[39]
		Having one’s achievements praised by others	[41]
		Feeling a sense of accomplishment	[41]
		Building ability and confidence	[41]
		Being able to develop and utilize one’s skills	[41]
	A workplace environment characterized by understanding symptoms and acceptance of diverse ways of working	Work experience with colleagues who have mental illness (“exposure experience”): Lack of this experience is significantly associated with negative perceptions	[29]
		Deepening understanding of symptoms in the workplace and shared knowledge about workplace management (explanation of common mental illness symptoms and responses)	[38]
		When symptoms arise, being provided with a calm environment at work and being understood and respected	[39]
		In some workplaces, advance notice of job/role changes and improved autism understanding among colleagues were cited as support measures	[33]
		When symptoms arise, being provided with a calm environment at work and being understood and respected	[39]
		A “noisy environment” is a common major difficulty for both autistic and non-autistic individuals	[33]
		Access to music	[41]
		Availability of safe and comfortable break spaces	[41]
Psychologically low-burden environment with predictability and autonomy	A workplace environment with predictability	Low task dispersion (minimal gap between job description and actual duties)	[36]
		High predictability of tasks	[36]
		Sense of consistent work availability	[41]
		Incorporate regular tripartite meetings to enable dialogue and knowledge transfer; clarify each party’s roles and responsibilities; explicitly outline necessary workplace accommodations (e.g., task adjustments)	[38]
		Support measures included advance notice of job content/role changes and improving colleagues’ understanding of autism	[33]
	A workplace environment that allows discretion and autonomous working	An environment designed with high job autonomy/control, clear roles and responsibilities, and no conflicting demands (role clarity)	[28]
		Having decision-making authority	[32]
		Ensuring autonomy	[32]
		For men: high decision-making authority and discretion regarding work approach, plus high job demands	[18]
		High job discretion: Low discretion significantly correlates with negative perceptions	[29]
		High job discretion (higher discretion reduces distress; this association strengthens with higher ADHD symptoms)	[31]
		Job management: High job discretion/control reduces both absenteeism and performance decline	[37]
		Ability to make decisions	[41]
		Ability to work at one’s own pace	[41]
		Workplace environment allowing satisfactory management of tasks	[41]
		IPS functions as an anchor in participants’ journey toward employment	[8]
		Self-employment or skilled manual labor	[37]
		The ES’s flexibility and availability, including outside of standard working hours, are pivotal in fostering trust, which is fundamental to building a robust relationship: “The trust built up in time… I could send her a message around the clock if there was something. That made me trust her more”	[8]
		Each person can contribute and feel a sense of responsibility	[41]
	A workplace environment with low psychological burden	Appropriate workload	[32]
		Low emotional labor	[32]
		For women: An environment with low emotional demands and fatigue	[18]
		For women: Low emotional demands and relatively little interpersonal interaction	[18]
		For men: Moderate interpersonal interaction	[18]
		Low quantitative demands	[36]
		Low emotional demands	[36]
		Low psychological burden (i.e., high psychological burden is a risk factor)	[30]
		Not excessively long working hours	[30]
		Workplaces without excessively long working hours (low working hours): Reduced working hours are also associated with improved job functionality	[37]
		Working hours are adjusted for each participant	[41]
		Balance between family life and cooperative activities	[41]
		Absence of pressure or stress	[41]
		Stimulating while allowing for seeking help from others	[41]
		Absence of ethical dilemmas	[30]
		Workplace environment free from bullying, verbal abuse, physical or sexual assault	[30]
		Low job demands	[31]
Multilayered support network environment	A workplace environment with supportive supervision	High-quality supervisor support environment	[28]
		Sense of solidarity with supervisors	[32]
		Proactive attitude of management	[39]
		Line managers recognize employees’ situations, understand and share their current work capacity and return-to-work intentions (leading to consideration for avoiding excessive burden)	[38]
		Positive relationships (especially between employee and supervisor) promote collaboration and are a success factor for return to work	[38]
		Supervisors who foster an atmosphere of understanding and tolerance toward mental illness	[41]
		Encouragement, guidance, and proactive feedback from supervisors	[41]
		In three-way meetings (HR representative, employee, supervisor), share objectives beforehand and ensure a “safe space for open discussion” during the meeting through clear facilitation (clarifying roles/responsibilities and discussing needs)	[38]
		Managerial support	[40]
	A workplace environment with coworker support and collaborative relationships	An environment with high-quality peer support	[28]
		High levels of workplace social support (support from supervisors + colleagues)	[29]
		High workplace social support	[30]
		Job Support: Workplaces with high support from colleagues and supervisors see reductions in both absenteeism and performance decline	[37]
		The presence of key mediators contributing to stress reduction (including support from supervisors and colleagues)	[32]
		Support from Colleagues and Supervisors	[36]
		Understanding and tolerance for individual differences among colleagues	[41]
		Mutual Support: Everyone has opportunities to be both a “receiver” and a “giver”	[41]
		Feeling accepted within work collaborations	[41]
		Rich social relationships	[36]
	A support environment involving family and community resources	Family Support	[39]
		Support from various people who understand my mental health issues, such as family, friends, and medical staff	[40]
		Social support is beneficial	[40]
	A workplace environment that provides a sense of safety and security	Not feeling a strong sense of loneliness	[41]
		Being able to talk to anyone	[41]
		Sense of community	[36]
		Annual trips	[41]
		The presence of role models in the workplace	[41]
		Ensuring safe and comfortable break spaces	[41]
	A workplace environment offering practical, life-based support	ESs emerge as providers of stability, offering practical support that ranges from securing housing, extended financial advice, ensuring mobility, or, in some cases, taking over as care coordinator.	[8]
Support environment grounded in interprofessional collaboration and utilization of systems	A workplace environment with access to employment support programs	Finding meaning in employment through the introduction of Individual Placement and Support (IPS)	[35]
		Employment support through participation in vocational support programs provided by District Mental Health (DMH) agencies	[35]
		Opportunities for social skills training through rehabilitation activities	[35]
		Provision of a comprehensive rehabilitation system with long-term support	[35]
		Existence of intervention programs providing opportunities and time for proactive communication	[32]
	A workplace environment with ongoing interprofessional support and coordinative involvement	High utilization of psychotherapy (especially among patients with personality disorders): Coping with internal distress improved workplace relationships	[35]
		Trust-based relationship and supportive involvement with Rehabilitation Coordinators (RC) (enhancing feelings of security, being understood, and motivation to participate)	[38]
		Supervisor involvement from the early stages of the return-to-work process, direct liaison and collaboration with the medical side (RC) (establishing “workplace-mediated support”)	[38]
		Development of shared tools such as manuals and worksheets (enabling shared frameworks and procedures among stakeholders)	[38]
		The RC’s role is formally positioned and supported within the medical institution (e.g., dedicated time slots). This enables sick leave/return-to-work support activities to proceed under a shared vision	[38]
		Mechanisms to identify disagreements or conflicts early and incorporate solutions into subsequent steps (visualizing disagreements and providing opportunities for adjustment)	[38]
		Large company size: Small companies are significantly associated with negative perceptions, while large workplaces have fewer negative perceptions and may offer a more favorable social environment in terms of organizational support resources	[29]
		Cooperative associations actively provide individual support	[41]
		Ongoing support from specialists	[40]
	A workplace environment with legal protections and use of social security systems	• Italian Law 68/99 (INAIL): Requires companies with 15 or more employees to employ persons with disabilities	[35]
		• Protected category: 12.4% of employees fell within this “protected category”	[35]
		• Law 104/92: Provides employment-related priority rights and special leave arrangements (applicable to 10.1% of participants)	[35]
		• Social security benefits (INPS pensions): 45.3% of the overall sample received INPS pensions, rising to 62.6% among individuals with psychotic disorders	[35]
		• Sickness allowances and pensions: Participants received financial support through sickness benefits and/or pensions	[41]

## Data Availability

No new data were created or analyzed in this study. Data sharing is not applicable.

## Supplementary Materials

The following supporting information can be downloaded at: https://www.mdpi.com/article/10.3390/nursrep16030101/s1, Table S1: Electronic search strategy for the PubMed database; PRISMA-ScR Checklist.

## Public Involvement Statement

No public involvement occurred in this study.

## Guidelines and Standards Statement

This manuscript was drafted against the PRISMA Extension (PRISMA-ScR) [27] for Scoping Reviews.
