# Supplementary material for: Psychosocial Workplace Environments Enabling Sustainable Employment for People with Mental Health Conditions: A Scoping Review
Source: Nurs Rep. 2026 Mar 17;16(3):101. doi: 10.3390/nursrep16030101 (PMC13028844; doi:10.3390/nursrep16030101)
Supplement: Supplementary file 1 [file nursrep-16-00101-s001.zip › Supplementary_Table_S1.pdf]

Supplementary Table S1. Full electronic search strategy for the PubMed database.

Search date: July 31, 2025 **Database:** PubMed

Search	Query
**#1**	("mental disorder" OR "mental illness" OR "mental health disorder" OR "psychiatric disorder" OR "schizophrenia" OR "depression" OR "bipolar disorder" OR "anxiety disorder" OR "post-traumatic stress disorder" OR "obsessive-compulsive disorder" OR "eating disorder" OR "substance use disorder" OR "autism" OR "Attention-deficit hyperactivity disorder (ADHD)")
**#2**	(employment OR "workplace integration" OR "workplace adaptation" OR "reasonable accommodations")
**#3**	("psychosocial environment of the workplace" OR "psychosocial conditions of the workplace" OR "psychosocial factors" OR "psychosocial factors in the workplace" OR "psychosocial support" OR "psychosocial interventions" OR "psychological workload")
**#4**	#1 AND #2 AND #3
**#5**	#4 AND (2003/01/01:2025/07/31[Date - Publication]) AND English [Language]

**Note: The final search (Step #5) yielded exactly 465 records on the original search date (July 31, 2025), corresponding to the number reported in the PRISMA flow diagram.*
